# Droplet motion on a wrinkled PDMS surface with a gradient structural length scale shorter than the droplet diameter†

**DOI:** 10.1039/d1ra09244h

**Published:** 2022-05-10

**Authors:** Yutaka Yamada, Kazuma Isobe, Akihiko Horibe

**Affiliations:** Graduate School of Natural Science and Technology, Okayama University Okayama 700-8530 Japan y.yamada@okayama-u.ac.jp +81 86 251 8046

## Abstract

Droplet transportation using a wettability gradient surface has attracted much attention owing to applications such as in microfluidic devices. A surface with a spatial structural gradient was prepared through a simple and cost-effective process even though understanding of droplet behavior on the structure was still limited. Here, we report impinging droplet motion on a gradient wrinkled surface. Surfaces were prepared through hard film deposition on soft pre-strained polydimethylsiloxane (PDMS) with a mask installed with a slit to control the amount of deposition, which is related to the wavelength of the wrinkles. Droplets were impinged with varying position with respect to the structure, and the droplet motion was observed in the direction away from the region under the slit. We found an asymmetric contact angle and alternate motion on both sides of the three-phase contact line during the motion according to the gradient of the wrinkle wavelength. These results may help not only to understand the behavior of droplet impingement on a gradient structural surface but also to further develop applications using directional droplet transfer.

## Abstract 2

Gradient wrinkled PDMS surfaces were fabricated through metal film deposition with installation of a mask and the motion of impinged droplets on the surfaces followed the structural gradient.

## Graphical abstract

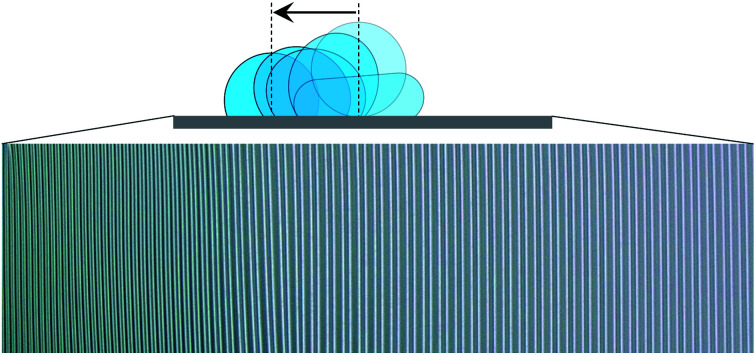

## 1. Introduction

A surface with a wettability gradient provides a promising means to transport a microliter-sized droplet,^1,2^ study cell adhesion characteristics^3^ and control the onset of nucleation.^4^ In particular, many applications of droplet motion such as microfluidic devices,^5^ chemical analysis,^6^ energy harvesting,^7^ water harvesting^8^ and condensation heat transfer^9^ have been investigated to enhance performance. The underlying physics of the wettability gradient is the surface energy gradient caused by nano/micro-scaled surface structures^10–12^ and chemicals grafted onto surfaces.^13,14^ Although some specially designed surfaces can be activated by external stimuli such as light irradiation^15^ and thermal input,^16^ most physical and chemical gradient surfaces show a wettability gradient without external stimulus. However, these have a drawback in the surface preparation procedures. For example, photolithography, microcontact printing and laser fabrication were used to prepare surface structures with extreme wettability based on the Cassie–Baxter or Wenzel wetting state,^10,11^ but this requires complex operations to prepare the surface. In addition, the accessibility of those fabrication devices is limited. This means that another method of providing a structural gradient surface is needed to enhance applications.

A wrinkled surface on an elastic substrate is a candidate for providing a nano- or micrometer-scaled structural surface without using microfabrication techniques. A typical preparation procedure is (1) creating a thin hard layer on the soft material under a pre-strain condition, and (2) releasing the applied strain.^17–27^ In recent years, investigations have used polydimethylsiloxane (PDMS) as a soft elastic substrate. The material of the thin hard layer depends on the preparation procedure. In particular, a silicate layer was formed through oxygen plasma irradiation^17,18^ or ultraviolet–ozone treatment,^19,20^ and several metals were applied through deposition.^21–23^ A characteristic dimension of a wrinkle is its wavelength, which ranges from the order of hundreds of nanometers to tens of micrometers, and is related to mechanical properties of the bilayer system and thickness of the thin layer.^22^ In addition, spatial control of the structure was achieved by installing a mechanical shutter or mask above the PDMS substrate during synthesis of the additional layer.^24–26^ For example, Lee *et al.* prepared stepwise wavelength patterns by covering a glass mask with various sizes to achieve the stepwise oxygen plasma dosage.^24^ Hiltl *et al.* installed an inclined mask on the PDMS substrate to generate the dosage gradient during plasma treatment and successfully fabricated a wavelength gradient ranging from 300 to 885 nm.^25^ Another approach by Parihar *et al.* used PDMS with a Young modulus gradient prepared by applying a temperature gradient during PDMS curing. Wrinkles with wavelength gradients ranging from 2500 to 3500 nm were produced through oxygen plasma treatment without a mask.^27^

The wettability characteristics of such surfaces have also been investigated. Experimental results showed that a longer wrinkle wavelength provided higher static contact angles along the direction parallel to the wrinkle structure, whereas a smaller contact angle was found in the perpendicular direction.^24,27^ In addition, Hiltl and Böker observed the behavior of a gently deposited droplet on a structural gradient surface.^26^ Motion perpendicular to the wrinkle direction was observed with a duration of a few tens of seconds. However, the understanding of impinging droplet behavior on a wrinkled surface is still limited.

In this study, we fabricated a structured surface by installing a mask with a slit during metal layer deposition. This procedure successfully prepared gradient wrinkled structures, and the wrinkle wavelength decreased with the distance from the region under the slit. In addition, the length scale of the gradient region was shorter than the droplet diameter (∼2.5 mm). The behavior of a dispensed droplet was characterized through side-view observation. The moving directions of droplets were divided in the region under the slit owing to the structural gradient.

## 2. Experimental methods

### Sample preparation

A PDMS substrate was used as a base material for wrinkle structure fabrication and was formed using the Sylgard 184 silicone elastomer kit (Dow Corning, US). The pre-polymer and curing agent were mixed in a weight ratio of 10 : 1 to form the base material, followed by curing at 70 °C for 2 h under vacuum. Then, the base material was cut into rectangles with a 20 mm width and 40 mm length. The typical thickness was 0.8 mm.

**Fig. 1:**
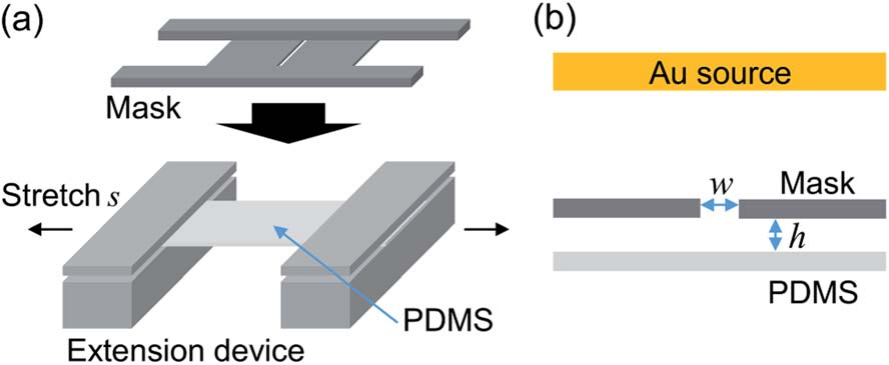
(a) Schematics of the extension device. (b) Illustration of the deposition region. The slit width of the installed mask and distance between the mask and PDMS surface are *w* and *h*, respectively.

Surface wrinkles were fabricated through deposition of a metal film on the PDMS surface under a pre-strain condition and following strain release. Previous investigations have revealed that the wrinkle wavelength changes with the Young's modulus and Poisson ratio of deposited materials and stretch ratio *s*, which is defined as the ratio of the substrate length after stretching to the initial length.^22,23^ In this study, the PDMS substrate was stretched using the extension device shown in Fig. 1a and the ratio *S* was set at 1.5 because further high stretch ratio induces the destruction of periodic structure. The metal mask with a slit was then installed on the device to induce the deposition gradient. Fig. 1b illustrates the cross section of the fabrication setup. The distance between the mask and PDMS substrate is defined as *h*, and the slit width is defined as *w*. Their values for samples S1–S3 are summarized in Table 1. Here, the distance *h* is defined in the unstretched condition. According to the Poisson effect, the substrate thickness is reduced during stretching, and it is estimated as 0.2 mm from the stretching ratio and Poisson ratio of PDMS.^28^ This means that 0.1 mm of additional separation was induced in all stretched samples assuming that the axis position does not change during stretching. A gold (Au) hard film was deposited on the stretched substrate by sputtering (IB-3, Eiko Co.) because the Au is a material that is difficult to oxidize. The ionic current was set at 4 mA under low pressure (∼20 Pa), and the sputtering duration was set at 4 min. The maximum thickness of the Au film was estimated as ∼20 nm under the slit. Lastly, the stretching was released to obtain a wrinkled substrate. In addition, samples with constant wrinkle wavelength were fabricated without a mask, and a planar PDMS substrate was prepared for wettability characterization.

**Table 1:** Sample preparation conditions

Sample no.	Au deposition	Stretch ratio *s* [—]	Height *h* [mm]	Width *w* [mm]
S1	Yes	1.5	1.0	2.0
S2	Yes	1.5	0.5	2.0
S3	Yes	1.5	0.5	1.0

### Sample characterization

Details of surface structures were characterized through laser microscopy (1LM15, Lasertec Co.). The local wavelength was determined as an average of at least five waves, and the measurement position was shifted by 0.1 mm to characterize the spatial variation of the wavelength.

The contact angle was characterized through the sessile droplet method. We gently deposited 3.5 μl of purified water (CPW-102, Advantec Co.) on the planar and constant wrinkled substrate. The droplet shape was captured from a horizontal orientation. For the wrinkled substrate, the measurement was conducted parallel to the wrinkle direction. The static contact angle was analyzed through in-house software using these images, and the obtained values were 115 ± 2° and 124 ± 4° for the planar and wrinkled substrate, respectively.

Droplet motion after contact with the gradient wrinkled structure was investigated by impinging with the purified water droplet with a volume of 7.7 ± 0.1 μl from above the substrate. The typical impact velocity of a droplet was 0.2 m s^−1^, which corresponds to the Weber number We = *ρDU*^2^/*γ* = 1.4, where *D*, *U*, *γ* and *ρ* are the diameter, impact velocity of the droplet, and surface tension and density of water, respectively. The droplet behavior was observed from the direction parallel to the wrinkle structure and recorded using a high-speed camera (HAS-U1, DITECT Co.) with a frame rate of 800 fps. The relation between the traveled distance Δ*x* after droplet impingement on the substrate and the deposited location was analyzed on the basis of the position of the droplet center. All experiments were conducted at least three times for each condition and substrate. The temperature of the surrounding air was 23 ± 2 °C, and the effect of evaporation was negligible because the duration of the three-phase contact line (TPCL) motion was less than 1 s.

## 3. Results and discussion

**Fig. 2:**
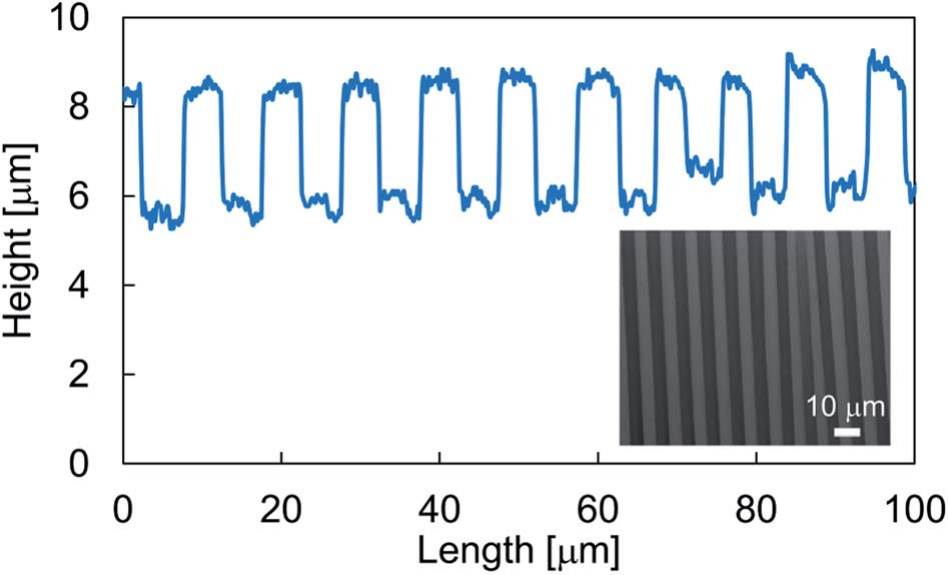
Typical cross sectional profile of a wrinkled structure. Inset panel showed a measured result of laser microscopy.

**Fig. 3:**
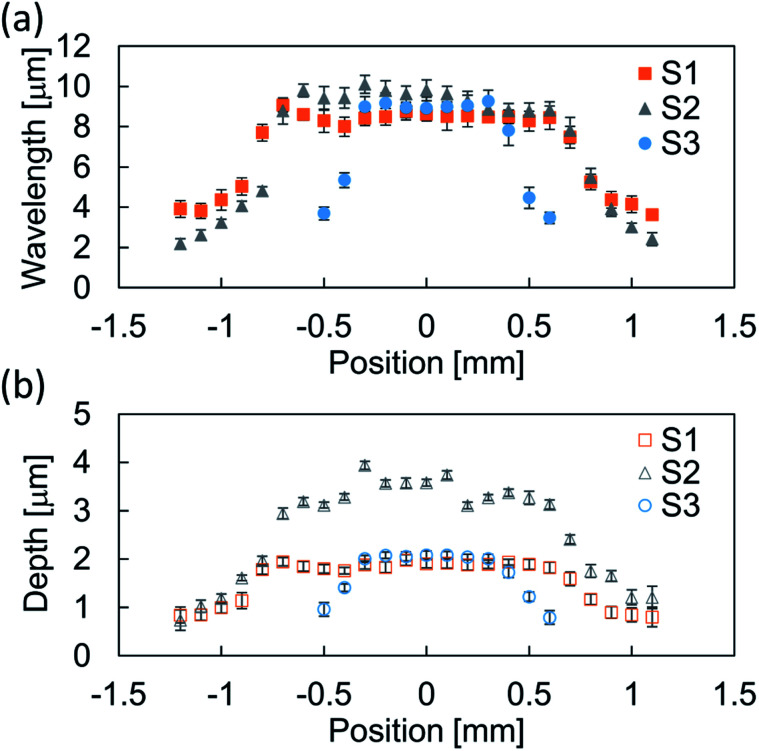
Relation between (a) position and wavelength, and (b) position and depth of samples S1–S3. The plotted data are the average of at least five waves, and the error bars indicate the standard deviation.

In our sample preparation procedure, the wrinkled structure appears after the pre-strain release. The wrinkles were then characterized to measure the wavelength. Fig. 2 showed a typical cross sectional profile of a wrinkled sample prepared without mask and inset panel showed a visualized surface structure. A periodic structure with a wavelength of ∼10 μm was obtained, and the structures had a height of 3 μm. To obtain the gradient wrinkled structure, we prepared samples with the mask installed above the stretched substrate. Fig. 3a shows the structural wavelength of each sample. Regions with constant structural wavelength were limited owing to the mask, and these were narrower than each slit width *w* shown in Table 1. These regions obviously corresponded to the area under the slit in the strained condition, and the wrinkled structure appeared after the strain release. Both ends of the constant-wavelength region were connected to the region with gradually decreasing wavelength because of the condition of the metal film deposition process. Sputtered Au particles were scattered by residual gas molecules, and the mean free path of the gas molecules was estimated as ∼350 μm at the operational pressure (∼20 Pa). This mean free path is not negligible with respect to *h*. This limited the deposition far from the slit. Consequently, a thickness gradient formed in the Au film and decreased with distance from the slit. As reported by Schedl *et al.*,^22^ the structural wavelength depends on the film thickness, and a thinner film forms a shorter wavelength. Therefore, the wavelength decreased according to distance from the region under the slit. In addition, the end of the gradient region connected to the region without Au deposition. The structural profile of S1 plotted in Fig. 3a shows a mild wavelength gradient compared with others. This is caused by the mild thickness gradient of the metal film due to the large separation between the mask and PDMS substrate, which provided the space to scatter Au particles. However, Au particles through the slit were deposited over a wide area. This induced a slightly narrower wavelength in the constant-wavelength region. The narrower slit induced the narrow constant-wavelength region S3 in Fig. 3a. Furthermore, the width of the gradient wrinkle region in all samples was estimated as less than 1 mm, which is narrower than the contact diameter of the impinging droplet. The depth of each wrinkled structure was also shown in Fig. 3b. Sample S2 showed deeper structure than other samples because the difference of preparation condition.

**Fig. 4:**
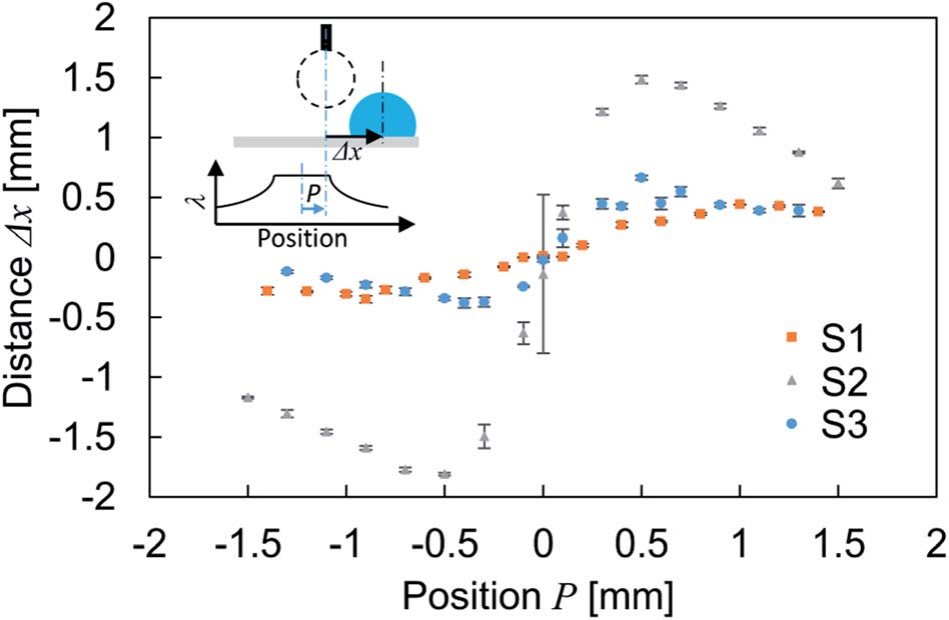
Relation between droplet deposition position *P* and transfer distance Δ*x*. The error bars show the standard deviation. The inset schematics show the experimental details. The bottom graph illustrates the relation between position and structural wavelength.

To characterize the droplet transfer performance on the wrinkled structures, the experiment described above was conducted by varying the position of droplet impingement. The parameter *P* indicates the relative position of the droplet center axis from the center of the constant-wavelength region at the moment of droplet impact on the substrate. Fig. 4 shows the transfer distance Δ*x* of the droplet with respect to the impinged position. This reveals the dependence of the droplet transfer direction on the impact position. The droplet transfer is directed to the side with a low structural wavelength because the hydrophobicity is degraded according to the diminished structural wavelength. The transfer distance increases and then approaches 0 according to the distance from *P* = 0. The result for sample S2 showed larger displacement than others. As one possibility, the transition of wetting state could be the reason of this difference. A previous report showed that the droplet motion follows the wetting characteristics in the uniform wetting state.^10^ On the other hand, the partial wetting transition induces motion directed opposite to the wettability gradient because the Wenzel state induces strong contact line pinning compared with the Cassie–Baxter state^29^ and it depends on the structural characteristics, impact velocity of the droplet and other parameters.^10,30–33^ However, in this study, a uniform wetting state is maintained during the bouncing because the direction of droplet motion follows the wettability characteristics and is assumed to be the Cassie–Baxter wetting state owing to the low impinging velocity. According to this, wetting transition can not explain the difference. Therefore, the larger spatial gradient of structural wavelength will be the reason of larger displacement.

**Fig. 5:**
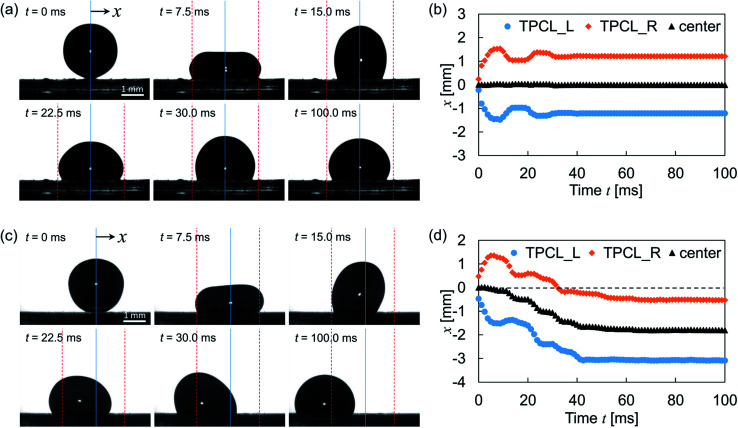
(a) Images captured at droplet impact on the wrinkleless PDMS surface and (b) corresponding time lapse profiles of the three-phase contact line positions and droplet center axis. (c) Images captured at droplet impact on sample S2 at *P* = −0.5 and (d) corresponding time lapse profiles of the three-phase contact line positions and droplet center axis. The letters “L” and “R” indicate “left” and “right,” respectively, in panels (b) and (d).

Time-resolved images after droplet impingement were analyzed to study the dynamic behavior of the droplet. Fig. 5a shows the bouncing behavior on the surface of wrinkleless PDMS (see Movie 1,† the movie plays at 1/40× speed). The vertical blue line indicates the droplet center axis at the moment of impact, and the red dashed lines indicate the TPCL position at maximum spreading on the surface. After the impingement, the droplet spread out symmetrically and reached the maximum diameter at time *t* = 7.5 ms. Then, the TPCL retracted symmetrically and repeated the expansion and retraction phases. The time-lapse profiles of TPCLs and droplet center positions are shown in Fig. 5b. After two obvious expansion and retraction phases, the equilibrium position is reached 40 ms after the deposition. During this vibration, the center axis of the droplet remained around the initial position, which means that the lateral directional motion was negligible. However, Fig. 5c shows typical droplet bouncing behavior on wrinkled and gradient structured surfaces (see Movie 2,† the movie plays at 1/40× speed). Here, the droplet was impinged on the left edge of the constant-wavelength region on sample S2, which means the left half of the droplet spread out on the gradient region of the wrinkled structure, whereas the right half was spread out on the constant-wavelength region. After impact, the maximum diameter was observed at *t* = 7.5 ms and was comparable with the result in Fig. 5a. However, the observed initial spreading phase was asymmetric, and the left TPCL was spread out 15% wider than the right TPCL. In addition, the left contact angle was smaller than the right one, and the liquid–gas interface of the droplet top was not parallel to the surface. After the spreading, the TPCL on the side with the larger structural wavelength retracted preferentially, whereas the TPCL on the side with the shorter wavelength was pinned on the surface. Then, advancing motion on the shorter-wavelength side followed. These motions were observed alternately within 100 ms as shown in Fig. 5d, and the droplet was directed to the side with the lower contact angle. Further motion of TPCL was not observed after 100 ms of the droplet deposition.

The dynamics of the droplet was analyzed on the basis of the uniform wetting state. During the expansion of the droplet impingement, kinetic energy is converted into interfacial energy with a certain energy loss due to viscous dissipation and deformation of the PDMS substrate.^34^ The energy stored as interfacial energy is then released and converted into kinetic energy again because the droplet deforms toward the equilibrium state. Although this series of energy conversions and droplet deformations is seen in Fig. 5a and b, the driving force of the lateral directional motion is absent on the wrinkleless substrate. This force was generated by the spatial variation of the surface structure along the direction perpendicular to the wrinkles. This force can be estimated as:^35^1
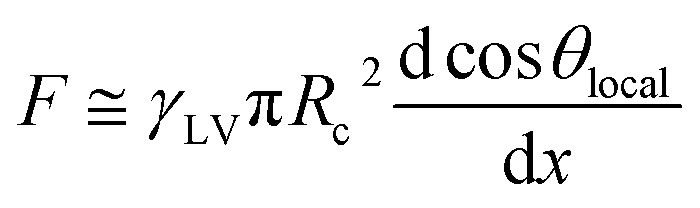
where *γ*_LV_ is the surface tension between the liquid and vapor phases, *R*_c_ is the contact radius of the droplet and *θ*_local_ is the local contact angle affected by the surface structure. In this study, the gradient region of the wrinkle structure was narrower than 1 mm, and the contact angle difference between both sides was estimated as ∼10°. This provided adequate driving force to allow lateral motion.

Although the lateral droplet motion was induced by the wettability difference as mentioned above, the mechanism of TPCL motion is discussed here because alternating motion of the TPCL was not observed with the gently deposited droplet.^26,36^ After impingement, both sides of the TPCL spread out on the surface, and eventually the retracting motion starts. Part of the energy released by the retraction is expended to form solid–vapor and liquid–vapor interfaces instead of a solid–liquid contact. This is quantitatively evaluated through the work of adhesion, which is described by the following equation:^37^

**Fig. 6:**
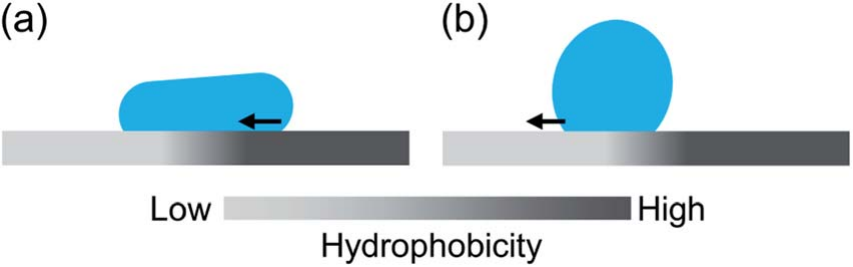
Schematics of the three-phase contact line motion in the (a) retracting phase and (b) expansion phase.

2 *W*_SL_ ≈ *γ*_LV_(1 + cos *θ*_local_)

where *W*_SL_ is the work of adhesion between the solid and liquid phases. Although *θ*_local_ depends on the impinged location, the side with the higher contact angle obviously showed lower *W*_SL_, which induced retraction on that side as shown in Fig. 6a. In the following expansion phase, the solid–liquid contact spreads out instead of the solid–vapor contact. The work of adhesion between the solid and vapor phases, *W*_SV_, is expressed in light of eqn (2) as:

3 *W*_SV_ ≈ *γ*_LV_(1 − cos *θ*_local_)

The lower-*θ*_local_ side is more favorable to advancing motion than the opposite side as shown in Fig. 6b. These TPCL motions occurred sequentially as long as the kinetic energy exceeded the required *W*_SL_ and *W*_SV_ for the motion, and the remaining energy was dissipated during droplet vibration owing to viscous dissipation and PDMS deformation.

**Fig. 7:**
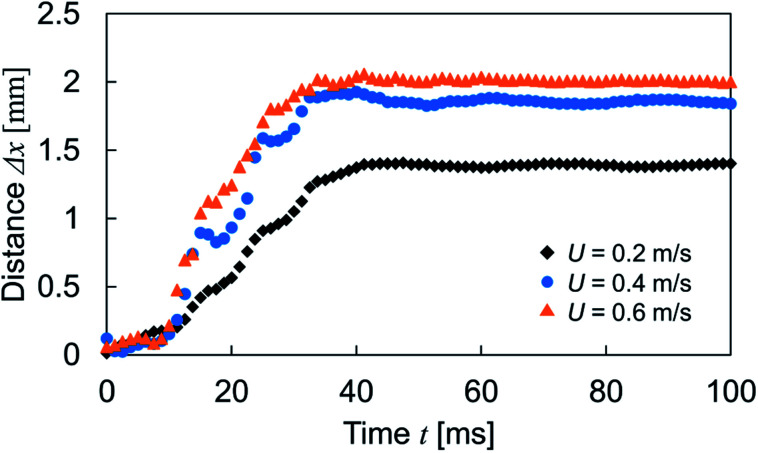
Relation between the transfer distance and impinging velocity as a function of time. Experiments were conducted with the sample S2 at *P* = 0.5.

In addition, we conducted the experiment with different droplet impinging velocity to discuss the relation with the transfer distance. The velocity was set at 0.2, 0.4 and 0.6 m s^−1^, and complete rebound was not observed in these velocity range. The result is shown in Fig. 7. Although obvious difference of transfer distance was not observed within ∼10 ms because of an initial spreading phase, the transfer distance increased as increase of the impinging velocity. The increase of the impinging velocity induces the increase of kinetic energy of droplet at the moment of impact. This reflects to the droplet deformation, converted interfacial energy and also to the energy expends to form the new interface according to eqn (2) and (3). Therefore, the increase of the transfer distance was observed and the result also indicated the absence of the wetting transition in this velocity range. The droplet and gradient structural dimensions will also affect to the transfer distance. As long as the dimension of the impinging droplet is much larger than that of the wrinkled structural wavelength and low We condition, the driving force for the lateral directional motion is generated by the macroscopic wettability difference due to the spatial variation of wrinkled structure shown as eqn (1). This is also for the situation when the dimension of the structural gradient region is wider than the dimension of droplet. And the work of adhesion required for the TPCL motion will follow eqn (2) and (3). According to these, smaller droplet with less amount of kinetic energy will show shorter transfer distance and expanding the wettability difference between both end of the gradient region is needed for the droplet motion on the surface with wider wettability gradient region.

## 4. Conclusions

We studied impinging droplet motion on micro-scaled wrinkles with a structural gradient. The wrinkle structure was prepared on a soft PDMS surface through metal deposition under a stretched condition and following release. The gradient wrinkle structure was successfully fabricated by installing a mask during the deposition. A wrinkle wavelength ranging from 2 to 10 μm was obtained while the width of the gradient region was less than 1 mm, which is narrower than the contact diameter of a droplet. A droplet impingement experiment showed the lateral droplet motion and the direction switched across the region with constant wavelength according to the wettability gradient. In addition, alternate motion of both sides of the TPCLs was observed during droplet bouncing owing to the difference in work of adhesion.

These results provide a useful way to create a wettability gradient surface without a complicated fabrication process such as microfabrication. In addition, the characteristics that droplets impinging around the hydrophobic region transfer to the more wettable region may help maintain the droplet-free region. According to present knowledge, reversing the direction of the wrinkle gradient would enable collection of droplets in the region between both gradient regions. Such wettability gradient surfaces may be helpful in microfluidics, water collection and condensation heat transfer.

## Conflicts of interest

The authors declare no competing financial interest.

## Supplementary Material

RA-012-D1RA09244H-s001

RA-012-D1RA09244H-s002
